# Prevalence and Correlates of Complementary and Alternative Medicine Use among Hypertensive Patients in Gondar Town, Ethiopia

**DOI:** 10.1155/2016/6987636

**Published:** 2016-10-23

**Authors:** Daniel Asfaw Erku, Abebe Basazn Mekuria

**Affiliations:** ^1^Department of Pharmaceutical Chemistry, School of Pharmacy, University of Gondar, Gondar, Ethiopia; ^2^Department of Pharmacology, School of Pharmacy, University of Gondar, Gondar, Ethiopia

## Abstract

*Background*. Complementary and alternative medicine (CAM) therapies are being widely used by hypertensive patients worldwide. However, evidences regarding CAM use by hypertensive patients in Ethiopia are limited. This study aimed at assessing prevalence and correlates of CAM use among hypertensive patients attending ambulatory clinic at Gondar University Referral Hospital (GURH), Ethiopia.* Methods*. A cross-sectional study was employed on 423 patients visiting GURH. Descriptive statistics and bivariate and multivariate logistic regression tools were used to analyze/come up with the prevalence and correlates of CAM use.* Results*. The prevalence of CAM use in our study was found to be 67.8% and herbal based medicine was the most commonly utilized CAM therapies. Majority of CAM users (70.2%) did not disclose CAM use for their physician. However, nearly half of CAM users (48.4%) were satisfied with the result of CAM use.* Conclusions*. The higher prevalence of CAM use among hypertensive patients coupled with a very low disclosure rate to their health care providers can have a marked potential to cause ineffective hypertensive management and adverse effects due to CAM use. Health care providers should be open to discussing the use of CAM with their patients as it will lead to better health outcome.

## 1. Introduction

Hypertension is one of the most common chronic cardiovascular diseases affecting up to 20% of the world's adult population [1]. It is estimated that up to 4.5% of the current global disease burden are due to hypertension [2]. Studies suggested that in Sub-Saharan Africa, overall hypertension prevalence is between 10 and 15% [3, 4]. As a result of the chronic nature of the disease and the complexities of treatment modalities, many hypertensive patients try to manage their disease through the use of different complementary and alternative medicine (CAM) practices.

Complementary and alternative medicine (CAM) is defined as a variety of ways including different medical and health care systems, various practices, and many products that are not treated as part of modern conventional medicine [5]. CAM therapies are being widely utilized around the globe by many of chronically ill patients owing to their high public acceptability [6, 7]. Chronic patients may prefer using CAM over modern medicine for many reasons including dissatisfaction with the conventional treatment, treatment related adverse effects encountered, and the perceived compatibility of CAM therapies with patients' values and spiritual beliefs [8]. Evidence suggests that CAM use is common among hypertensive patients (8%–40%) while most of them use it alongside conventional medical treatments [9, 10]. Among these, herbal products are the most commonly used CAM modalities [11]. Currently, the frequency of CAM utilization is increasing worldwide and is well documented in many African countries. Studies reported that about 80% of chronic patients in Morocco and 63.9% of chronic patients in India used herbal medicines for the management of their illnesses. The variation in prevalence across different countries may be attributed to the differences in research design and definitions of CAM employed by different researchers. Studies also showed that age, sex, level of education a, and presence of comorbidity have a higher correlation with the use of CAM [12]. Conducting researches on the prevalence of CAM use and disclosure rate to healthcare professionals helps to pinpoint how patients find it important and to discuss their CAM use with health care providers, which could eventually affect effectiveness of the health care delivered. CAM use by chronically ill patients in Ethiopia is not only common but also culturally acknowledged. However, there is still a paucity of data regarding prevalence and associated factors of CAM use among Ethiopian hypertensive patients. This study aimed to investigate the prevalence and associated factors of CAM use among hypertensive patients in Gondar, Ethiopia.

## 2. Materials and Methods

### 2.1. Study Design and Setting

This was a cross-sectional survey of hypertensive patients attending ambulatory clinic at Gondar University Referral Hospital (GURH). The study was conducted from April to June 2016. GURH is located in Gondar town, North West Ethiopia, 727 km away the capital city of Ethiopia. It is one of the oldest and pioneering referral teaching hospitals in Ethiopia with a range of specialists including a chronic disease illness follow-up ambulatory clinic, which currently provides free service for more than 15,000 chronic cardiovascular patients on outpatient level annually.

### 2.2. Population and Sampling

A convenience sample of adult hypertensive patients attending GURH ambulatory clinic were invited. 423 patients were enrolled in study within two-month data collection period. The inclusion criteria were adult (≥18 year age) hypertensive patients regardless of stage and time since diagnosis, who started taking medication for reduction of blood pressure and visited hypertension outpatient ambulatory clinic from April to June 2016. Those patients who lack understanding of oral Amharic language and had severe physical or psychological problems as well as those who refused to participate were excluded. The two-month follow-up period was chosen for data collection to avoid duplication of the cases as patients return to the ambulatory clinic every two months.

### 2.3. Data Collection Method and Survey Instrument

Data collection was performed by five well trained nurses who were working at ambulatory clinic through interviewer-administered questionnaire. The data collection tool was developed after a through literature review of the published studies [12–17] and prepared in English. This was translated to Amharic which is then translated back to English in order to ensure that the translated version gives the proper meaning. The final tool was pretested on 25 hypertensive patients who were not included in the final analysis and relevant modifications were instituted before the commencement of actual data collection. The final questionnaire constitutes 21 items that were divided into two main parts. Sociodemographic and disease related data including age, gender, level of educational, monthly income, and duration of hypertension were documented. The second part aimed to assess the level of CAM use, information source, and discussion with physicians about CAM use. Patients were given five categories to choose from and told that more than one choice is possible. The type of CAM modalities was classified as biological based therapies (herbal medicine, honey, animal products diet, and natural products (vitamins and minerals)), manipulative and body based therapies (exercise, massage, and relaxation), and mind/body intervention, spiritual healing which includes prayers, lighting candles, consuming holy water such as “Tsebel” (a type of holy water used by orthodox Christians), and fasting (abstinence from any food or drink) and listening to music.

### 2.4. Statistical Analysis

The collected data were entered into and analyzed using the Statistical Package for the Social Sciences (SPSS) software version 21.0 for Windows. Frequencies and percentages were used to express different variables. The baseline characteristics of CAM users and nonusers were compared by using Pearson's chi-square test. Both bivariant and multivariant logistic regression methods were also employed to identify determinants of CAM use. The results were adjusted for patients' demographic and clinical characteristics. Odds ratio (OR) with 95% confidence interval (95% CI) were also computed along with corresponding *p* value (*p* < 0.05).

### 2.5. Ethical Clearance

This study was approved by the ethical committee of University of Gondar. Informed consent was also secured from the participants prior to the data collection and participants' information obtained was kept anonymous.

## 3. Results

### 3.1. Sociodemographic and Clinical Characteristics

Out of 423 hypertensive patients invited to participate, 412 of them completed the survey giving a response rate of 97.39%. More than half of respondents (59.5%) were female and married (69.8%). Majority of respondents were orthodox Christians (85.9%) and 65.3% of them had <100 USD average monthly income. Around two-third (66.9%) of respondents had a family history of hypertension and 36.1% of them develop complication. Other sociodemographic and clinical characteristics of respondents are shown in Table 1.

### 3.2. Patterns of CAM Utilization

The use of CAM was reported by 279 (67.8%) of respondents. The type of CAM modalities was classified as biological based therapies, manipulative and body based therapies, and mind/body intervention. Accordingly, the most common biological based CAM preparations reported by respondents were herbal based medicine (67.5%), honey (44.1%), animal products (34.8%), diet (33.7%), and natural products (vitamins and minerals) (56.3%). Other CAM modalities reported were manipulative and body based therapies (exercise, 50.9%), massage (26.5%), and relaxation (8.6%) as well as mind/body intervention like fasting (22.9%). The pattern and types of various CAM used by respondents are depicted in Table 2.

The prevalence and characteristics of CAM use are summarized in Table 3. Among CAM users, 210 (75.3%) used CAM as complementary treatment along with the conventional medicine. Traditional herbalist was (37.6%) the most commonly cited source of recommendation about CAM followed by families and friends (29%). Most of the CAM users (27.6%) cite dissatisfaction with conventional medicine as the most common reason for using CAM. Similarly, the most common reason for not using CAM among nonusers was due to being afraid of side effect (39.8%) followed by lack of belief in its effectiveness (22.7%). Majority of CAM users (70.2%) did not disclose CAM use for their physician due to fear of their health providers (68.8%) and lack of sufficient information about CAM (22.9%). About 79.9% of CAM users have not experienced any apparent side effects, and nearly half of them (48.4%) were satisfied with the result of CAM use.

### 3.3. Determinants CAM Use

The determinants of CAM, obtained by using logistic regression analysis, are presented in Table 4. The odds of CAM use among rural residents are 2.14 times higher than urban residents (AOR: 2.14, 95% CI: 1.162, 5.153). Men patients were 2.23 times as likely to use CAM (AOR: 2.23, 95% CI: 1.173, 6.142) than female patients. Patients who had no formal education were 3.140 times more likely to use CAM than those who attended university (AOR: 3.140, 95% CI: 1.374–5.245). Patients who had average monthly income of less than 100 USD were 2.076 times more likely to use CAM than those who had average monthly income of greater than 150 USD (AOR: 2.076, 95% CI: 1.322–7.566). The odds for CAM use among patients who develop complications were 3.236 times higher than in patients without HTN complications (AOR: 3.236, 95% CI: 1.171–5.113). The CAM user among patients had family history of HTN being 2.673 higher than in patients without family history of HTN (AOR: 2.673, 95% CI: 1.583–6.716). However, there is no significant association found between CAM use and age, religion, employment status, BMIs, and duration of disease in both the bivariant and multivariant logistic regression analysis.

## 4. Discussion

Currently, CAM therapies are being widely used around the globe by many of chronically ill patients including hypertension [6, 7]. This study tried to assess the prevalence and correlation of CAM use among hypertensive patients in Ethiopia. The prevalence of CAM use found in our study (67.8%) is comparable with a study done in India (63.9%) [14], but it is higher compared with the studies done Nigeria 29% [15], Ghana 19.5% [16], South Africa 21% [17], USA 40% [18], and Australia 48.5% [19]. However, studies done in Morocco 80% [13] and Palestine 85.7% report a higher prevalence of CAM use [20]. The variations in the prevalence of CAM use across different countries might be due to the variations in sociocultural background, accessibility of modern medical practice, and perceptions of the importance of CAM. Among the CAM users, 67.5% of respondents utilize herbal based medicine which is comparable with the study done in Nigeria 63% [15]. The high prevalence of CAM use in general and herbal based medicines in particular could be due to the fact that the tradition and culture in Ethiopia encourage CAM use. CAM use in Sub-Saharan Africa has been linked with cultural beliefs and advice from family and friends [21]. Furthermore, Ethiopia is endowed with a rich and diverse flora that comprised a foundation for primary health care [22]. In Sub-Saharan African countries like Ethiopia, CAM use is augmented by the presence of many traditional medicine practitioners. Even though herbal products are shown to be effective in reducing the blood pressure [23], they contain toxic substances which could affect the health of the patient and render the modern drugs ineffective. Furthermore, spiritual healing (fasting, prayer, and Tsebel) was used by a considerable percentage of CAM users (88.2%), which is consistent with a previous study [24]. A common practice to all religions in Ethiopia is the incorporation of religious convictions in daily practices, with prayer and fasting being an integral piece of the culture. Most of the spiritual practices have the advantage of being safe, cheap, and easy to use though their effectiveness is not certain. In our study, rural residence, higher educational status, male gender, low average monthly income, development of complications, and having family history of hypertension were significantly associated with CAM use. Patients with a higher educational status may be more likely to explore for other therapies and ways to muddle through with the disease state and treatment effects [25].

In our study, traditional herbalist was the most commonly cited source of recommendation about CAM followed by families and friends. In contrast, medical practitioners were the least information source for CAM use (4.6%). This result is also similar to the study done in Germany, where the most prominent sources of information for CAM choice were outside the medical scheme and included families, relatives, and friends (49%) [26]. Disclosure of CAM use to health care practitioners was not practiced by majority (70.2%) of CAM users. A similar finding was reported by cancer patients in Ethiopia [27]. To prevent abuse of CAM, health care providers should give emphasis to patient CAM use. The negative attitude of health care providers to CAM practices and their prominence on scientific evidence may discourage patients from sharing information about their CAM use. As the lack of communication may lead to a negative overall health status, physicians should acknowledge patients' CAM use and encouraging active conversation for the proper use of CAM.

As a limitation, the results found regarding CAM use may not be representative because the present study is conducted in only one referral hospital. A larger-scale and multicentered survey that includes more diverse participants is needed to provide more accurate findings.

## 5. Conclusions

In summary, this study revealed that CAM use is prevalent among hypertensive patients, herbal based medicine being the most commonly used. Health care providers' role in orienting CAM use in hypertensive patients was negligible as patients relied mainly on traditional herbalist followed by family and friends for the choice of CAM and were less likely to disclose CAM use to health care practitioners. The patients' higher magnitude of CAM use along with very low disclosure rate to their health care providers can have a marked potential to cause ineffective disease state management and adverse effects due to CAM use. Health care providers should be open to discuss the use of CAM with their patients as it will lead to better health outcome. With the increasing importance of CAM in modern health care, medical, and nursing education should include information about CAM practices.

## Figures and Tables

**Table 1:** Sociodemographic and clinical characteristics of respondents, Gondar, 2016 (*N* = 412).

Variables	Overall (*n* = 412)	CAM users (*n* = 279)	Non-CAM users (*n* = 133)	*p* value
*Age *				0.124
Mean (SD)	57.32 ± 10.57	60.6 (10.6)	53.6 (10.6)	
*Sex *				0.011^*∗*^
Male	167 (40.5%)	115 (41.2%)	52 (39.1%)	
Female	245 (59.5%)	164 (58.8%)	81 (60.9%)	
*Residence *				0.031^*∗*^
Urban	225 (54.6%)	106 (37.9)	119 (89.5%)	
Rural	187 (45.4%)	173 (62.1%)	14 (10.5%)	
*Educational status*				0.002^*∗*^
No formal education	142 (34.5%)	123 (44.1%)	19 (14.3%)	
Primary	118 (28.6%)	54 (19.3%)	64 (48.2%)	
Secondary	83 (20.1%)	50 (17.9%)	33 (24.8%)	
University	69 (16.7%)	34 (12.3%)	35 (26.3%)	
*BMI (kg/m* ^*2*^)				0.229
Mean (SD)	28.4 (5.4)	29.8 (5.2)	26.9 (5.6)	
*Marital status*				0.431
Single	17 (4.1%)	9 (3.2%)	8 (6%)	
Married	283 (69.8%)	222 (79.5%)	61 (45.9%)	
Widowed	97 (23.5%)	36 (12.9%)	61 (45.9%)	
Divorced/separated	15 (3.6%)	12 (4.3%)	3 (2.2%)	
*Average monthly income*				0.013^*∗*^
<100 USD	269 (65.3%)	219 (78.5%)	50 (37.6%)	
100–150 USD	83 (20.1%)	55 (19.7%)	28 (21.1%)	
>150 USD	60 (14.6%)	5 (1.7%)	55 (41.3%)	
*Employment status*				0.273
Self-employed	60 (14.6%)	40 (14.3%)	20 (15.1%)	
Government-employed	107 (26%)	70 (25.1%)	37 (27.8%)	
Unemployed	245 (59.4%)	169 (60.6%)	76 (57.1%)	
*Religion*				0.328
Orthodox	354 (85.9%)	274 (98.2%)	80 (60.1%)	
Muslim	38 (9.2%)	3 (1.1%)	35 (26.3%)	
Others^*∗∗*^	20 (4.8%)	2 (0.7%)	18 (13.6%)	
*Duration of disease*				0.432
≤5 years	115 (27.9%)	32 (11.5%)	83 (62.4%)	
>5 years	297 (72.1%)	247 (88.5%)	50 (37.6%)	
*Presence of HTN complications*				0.004^*∗*^
No	168 (40.8%)	83 (29.8%)	85 (63.9%)	
Yes	244 (59.2%)	196 (70.2%)	48 (36.1%)	
*Family history of HTN*				0.001^*∗*^
No	102 (24.5%)	58 (20.8%)	44 (32.3%)	
Yes	310 (75.5%)	221 (79.2%)	89 (66.9%)	

^*∗*^Significant association (*p* value less than 0.05). ^*∗∗*^Jehovah Witnesses, Catholic, and Protestant.

HTN: hypertension; CAM: complementary and alternative medicine.

**Table 2:** Types of CAM utilized by respondents, GURH, Ethiopia, 2016 (*N* = 279).

Type of CAM	Frequency (%)
*Biological based therapies* ^*∗*^	
Herbal based medicine	189 (67.5%)
Honey	123 (44.1%)
Animal products	97 (34.8%)
Diet	94 (33.7%)
Natural products (vitamins and minerals)	157 (56.3%)
*Manipulative and body based therapies* ^*∗*^	
Exercise	159 (50.9%)
Massage	74 (26.5%)
Relaxation	24 (8.6%)
*Mind/body intervention* ^*∗*^	
Fasting	64 (22.9%)
Prayers	85 (30.5%)
Tsebel (holy water used by orthodox Christians)	97 (34.8%)
Listening to music	19 (6.7%)

^*∗*^More than one choice is possible.

**Table 3:** Prevalence and characteristics of CAM use among respondents, GURH, Ethiopia, 2016 (*N* = 412).

Variables	Frequency (%)
*CAM use since diagnosis*	
No	133 (32.2%)
Yes	279 (67.8%)
*How do you use CAM?*	
Complementary to modern medicine	210 (75.3%)
Alternative to modern medicine	22 (7.9%)
Both	47 (16.8%)
*Who recommended you to use CAM?*	
Traditional herbalist	105 (37.6%)
Families and friends	85 (30.5%)
Patients used CAM	55 (19.7%)
Health care professionals	13 (4.6%)
Others^*∗*^	21 (7.5%)
*Reasons for CAM use*	
The tradition in resident area encourages CAM use	50 (17.9%)
Belief in advantages of CAM	73 (26.2%)
Accessibility (availability)	45 (16.1%)
For the treatment of other medical conditions	20 (7.2%)
Dissatisfaction with modern medicine	77 (27.6%)
Others^*∗∗*^	14 (5.0%)
*Reasons for not using CAM among nonusers*	
Additional burden	21 (15.7%)
Afraid of side effect	53 (39.8%)
The doctor did not recommend (prescribe) it	29 (21.8%)
Lack of belief in its effectiveness	30 (22.7%)
*Disclosure for health care professionals (HCPs)*	
No	196 (70.2%)
Yes	83 (29.8%)
*Reason for not disclosing*	
Fear of response of HCPs	192 (68.8%)
Not necessary	23 (8.3%)
Insufficient information of CAM	64 (22.9%)
*Apparent side effects*	
No	223 (79.9%)
Yes	56 (20.1%)
*Satisfaction*	
Satisfied	135 (48.4%)
Average	105 (37.6%)
Dissatisfied	39 (14%)

^*∗*^Religious teachers and social media; ^*∗∗*^no clinic near the house.

HCPs: Health care professionals.

**Table 4:** Predictors of CAM use among respondents using regression analysis, GURH, Ethiopia, 2016 (*N* = 412).

Variables	CAM use		Logistic regression analysis	
	Yes	No	COR (95% CI)	AOR (95% CI)
*Residence*				
Urban	106	119	1	1
Rural	173	14	3.34 (1.804,5.063)	2.14 (1.162, 5.153)
*Sex*				
Male	115	52	2.45 (1.813,4.173)	2.23 (1.173, 6.142)
Female	164	81	1	1
*Educational status*				
No formal education	123	19	1	1
Primary	54	64	1.698 (1.875–3.295)	1.993 (0.404–3.224)
Secondary	50	33	1.1921 (1.025–2.171)	2.141 (1.872–5.217)
University	34	35	3.124 (1.737–5.872)	3.140 (1.374–5.245)
*Average monthly income*				
<100	219	50	3.179 (1.944–5.566)	2.076 (1.322–7.566)
100–150	55	28	2.139 (1.064–4.298)	1.597 (1.142–5.422)
>150	5	55	1	1
*Presence of HTN complications*				
No	83	85	1	1
Yes	196	48	4.593 (1.261–1.348)	3.236 (1.171–5.113)
*Family history of HTN*				
No	58	44	1	1
Yes	221	89	2.942 (1.603–6.552)	2.673 (1.583–6.716)

## Abbreviations

CAM: Complementary and alternative medicine
CI: Confidence interval
GURH: Gondar University Referral Hospital
HTN: Hypertension
OR: Odds ratio
SPSS: Statistical Package for the Social Sciences.
